# Local Spatial and Temporal Processes of Influenza in Pennsylvania, USA: 2003–2009

**DOI:** 10.1371/journal.pone.0034245

**Published:** 2012-03-28

**Authors:** James H. Stark, Ravi Sharma, Stephen Ostroff, Derek A. T. Cummings, Bard Ermentrout, Samuel Stebbins, Donald S. Burke, Stephen R. Wisniewski

**Affiliations:** 1 New York City Department of Health and Mental Hygiene, New York, New York, United States of America; 2 Department of Behavioral and Community Health Sciences, Graduate School of Public Health, University of Pittsburgh, Pittsburgh, Pennsylvania, United States of America; 3 Bureau of Epidemiology, Pennsylvania Department of Health, Harrisburg, Pennsylvania, United States of America; 4 Department of Epidemiology, Bloomberg School of Public Health, Johns Hopkins University, Baltimore, Maryland, United States of America; 5 Department of Mathematics, School of Arts and Sciences, University of Pittsburgh, Pittsburgh, Pennsylvania, United States of America; 6 Department of Epidemiology, Graduate School of Public Health, University of Pittsburgh, Pittsburgh, Pennsylvania, United States of America; Northeastern University, United States of America

## Abstract

**Background:**

Influenza is a contagious respiratory disease responsible for annual seasonal epidemics in temperate climates. An understanding of how influenza spreads geographically and temporally within regions could result in improved public health prevention programs. The purpose of this study was to summarize the spatial and temporal spread of influenza using data obtained from the Pennsylvania Department of Health's influenza surveillance system.

**Methodology and Findings:**

We evaluated the spatial and temporal patterns of laboratory-confirmed influenza cases in Pennsylvania, United States from six influenza seasons (2003–2009). Using a test of spatial autocorrelation, local clusters of elevated risk were identified in the South Central region of the state. Multivariable logistic regression indicated that lower monthly precipitation levels during the influenza season (OR = 0.52, 95% CI: 0.28, 0.94), fewer residents over age 64 (OR = 0.27, 95% CI: 0.10, 0.73) and fewer residents with more than a high school education (OR = 0.76, 95% CI: 0.61, 0.95) were significantly associated with membership in this cluster. In addition, time series analysis revealed a temporal lag in the peak timing of the influenza B epidemic compared to the influenza A epidemic.

**Conclusions:**

These findings illustrate a distinct spatial cluster of cases in the South Central region of Pennsylvania. Further examination of the regional transmission dynamics within these clusters may be useful in planning public health influenza prevention programs.

## Introduction

Each year significant resources are expended by public health officials and health care providers to prevent and mitigate influenza epidemics. Decisions on how to allocate resources for prevention programs and vaccination campaigns often rely on macro-level information and recommendations without regard to spatially and temporally explicit illness patterns. Knowledge of local geographic distribution would likely improve the ability of public health agencies to allocate human and material resources and allow improved targeting and timing of prevention and control measures.

Despite the need for community-based influenza analyses, few studies have explored the spatial and temporal dynamics of incidence on a narrow geographic scale (state or county) appropriate to inform local public health officials [1], [2], [3]. An analysis of influenza hospitalizations in Colorado, United States, noted differences in regional peak timing, influenza B temporality, and age group-specific rates for influenza B hospitalizations [3]. Crighton et al. noted spatial heterogeneity in pneumonia and influenza hospitalization rates within urban and rural counties across age groups in Ontario, Canada [2]. These analyses help to explain the regional spatiotemporal patterns of influenza within a state or province; however, hospitalization data used for these analyses often represents estimates of severe morbidity and may not accurately reflect timing of either peak influenza activity or the true incidence patterns.

Further evaluations of seasonal transmission dynamics have concentrated on broad geographic scales such as a country or continent, often using data aggregated at larger spatial scales [4], [5], [6], [7], [8], [9], [10], [11], [12]. Analyses conducted at smaller spatial scales may capture unique local trends in disease structure potentially concealed in analyses of data aggregated at large scales. The details of local spatial dynamics may reveal the effect of population structure or environmental factors on influenza incidence.

In 2003, a new Pennsylvania law led to mandatory influenza case reporting from all laboratories, providers and hospitals resulting in a detailed spatio-temporal data source not previously available. As a result, a new opportunity exists to assess the local trends in disease. We conducted an exploratory ecological study evaluating the spatial and temporal patterns of laboratory-confirmed influenza cases in Pennsylvania from six consecutive influenza seasons (2003–2009) using Pennsylvania's National Electronic Disease Surveillance System (PA-NEDSS). Specifically, we assessed spatial incidence clusters, predictors, and temporal variation. Pennsylvania's diverse geography and population structure make it a unique locale to evaluate these dynamics.

## Results

All 67 counties in Pennsylvania reported at least one case of laboratory-confirmed influenza over the six year period and a total of 57598 cases were reported to the Pennsylvania Department of Health during the study period (Table 1). The greatest number of reported cases occurred during the 2007/08 influenza season; while the 2006/07 season reported the fewest. Co-circulation of influenza A and B occurred during all 6 seasons; however in 2003/04, the percentage of reported typed viruses that were B was approximately 1%. This is in contrast to the 2008/09 season in which 42% of all typed viruses were B; the most in any of the 6 seasons.

**Table 1:** Characteristics of reported influenza cases in Pennsylvania, USA, 2003–2008 influenza seasons.

	Influenza season, no. (%)													
Variable	Cumulative		2003–2004		2004–2005		2005–2006		2006–2007		2007–2008		2008–2009	
Number of Cases	57598		8836	15.34%	11293	19.61%	8717	15.13%	3997	6.94%	16657	28.92%	8098	14.06%
Flu Type														
A	35307	71.33%	5670	64.17%	8557	75.77%	6547	75.11%	3264	81.66%	11269	67.65%	4550	56.19%
B	8169	16.50%	59	0.67%	1369	12.12%	1692	19.41%	563	14.09%	4486	26.93%	3404	42.04%
Unknown	6023	12.17%	3107	35.16%	1367	12.10%	477	5.47%	170	4.25%	902	5.42%	144	1.78%
Gender														
Male	23057	46.58%	4151	46.98%	5154	45.64%	4098	47.02%	1937	48.46%	7717	46.33%	3881	47.93%
Female	26395	53.32%	4683	53.00%	6135	54.33%	4616	52.96%	2055	51.41%	8906	53.47%	4196	51.82%
Unknown	47	0.09%	2	0.02%	4	0.04%	2	0.02%	5	0.13%	34	0.20%	21	0.26%
Age* *														
Mean	34		31		45		33		27		35		22	
Median	27		19		46		24		19		31		17	
Under 5 years	9396	16.32%	2871	32.50%	1253	11.10%	1338	15.35%	684	17.11%	2077	12.48%	1173	14.49%
5 to 19 years	14461	25.12%	1626	18.41%	1817	16.09%	2632	30.19%	1348	33.73%	3562	21.41%	3476	42.92%
20 to 44 years	14929	25.93%	1499	16.97%	2483	21.99%	1942	22.28%	1063	26.59%	5520	33.17%	2422	29.91%
45 to 64 years	8479	14.73%	866	9.80%	2216	19.62%	1224	14.04%	499	12.48%	2918	17.54%	756	9.34%
65 years and over	10314	17.91%	1972	22.32%	3524	31.21%	1581	18.14%	403	10.08%	2563	15.40%	271	3.35%

* Nineteen subjects have missing date of birth.

**Figure 1:**
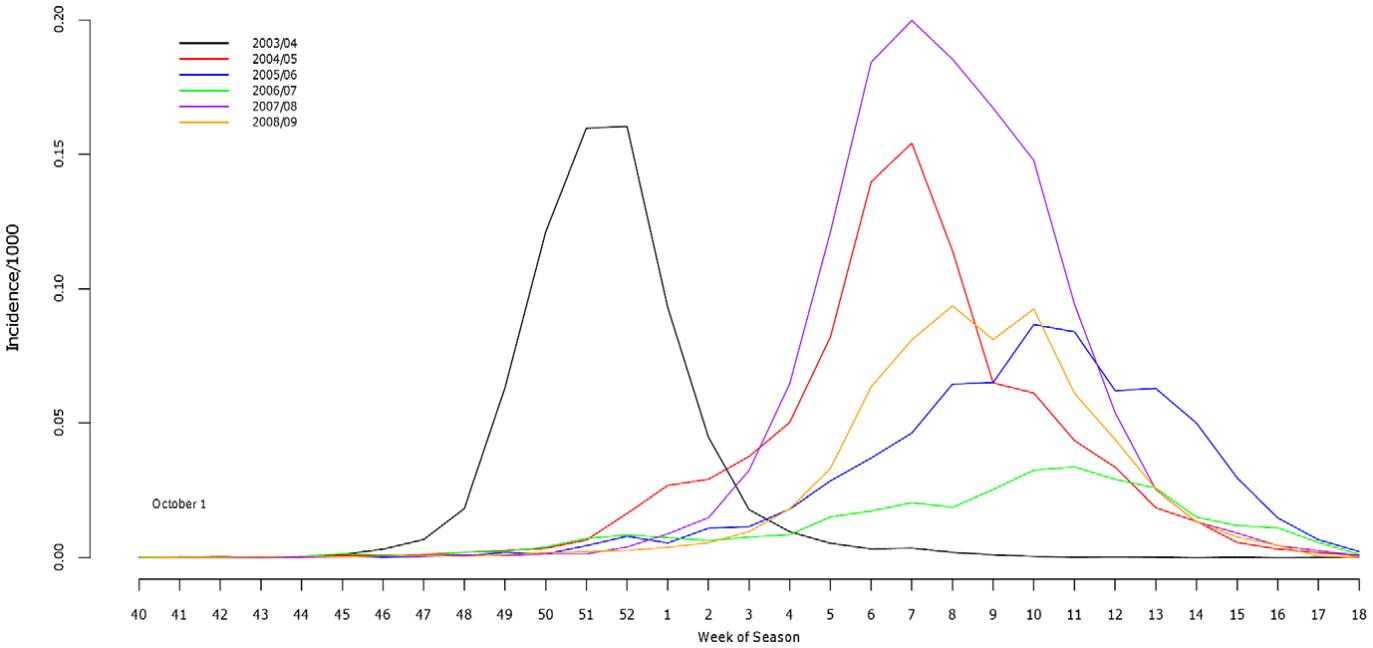
Weekly time series of influenza incidence in Pennsylvania (sum of all counties) superimposed for 6 influenza seasons (2003–2009).

**Figure 2:**
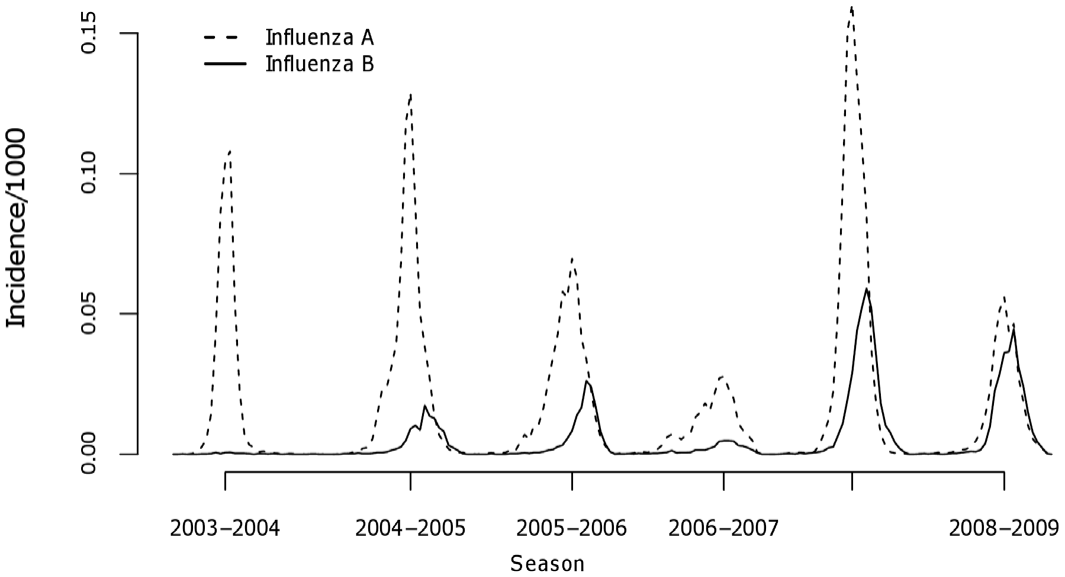
Weekly incidence by influenza type in Pennsylvania for 6 consecutive seasons (2003–2009).

In the time series of reported influenza cases, only the 2003/04 season peaked prior to January 1 (Figure 1). Each of the consecutive seasons peaked post-January 1 and the 2007/08 season had the greatest weekly magnitude. The 2006/07 season exhibited the latest weekly peaks. Season 2003/04 experienced the shortest peak epidemic length (2.33 weeks) which was significantly shorter than the other 5 seasons (Table 2). Seasons 2004/05, 2007/08, and 2008/09 had confidence intervals and point estimates that overlapped indicating that durations were not different. Details of individual model fit including standard errors are provided in Figure S1. Evaluation of the time series stratified by influenza type yielded two important observations reflecting the subtype epidemics (Figure 2). First, peak incidence of influenza B epidemics lagged influenza A epidemics by approximately 3 weeks (mean = 2.75). Second, the decline in weekly cases coincided for both influenza A and B time series in each of the seasons reporting significant influenza B cases even as surveillance systems were maintained.

**Table 2:** Epidemic width estimates and confidence intervals.

Season	σ*	95% Confidence Interval
Season 2003/04	2.33	2.26, 2.39
Season 2004/05	3.6	3.2, 4.0
Season 2005/06	4.89	4.58, 5.2
Season 2006/07	5.9	5.19, 6.60
Season 2007/08	3.72	3.56, 3.87
Season 2008/09	3.82	3.64, 4.01

* Sigma measures the epidemic length.

**Figure 3:**
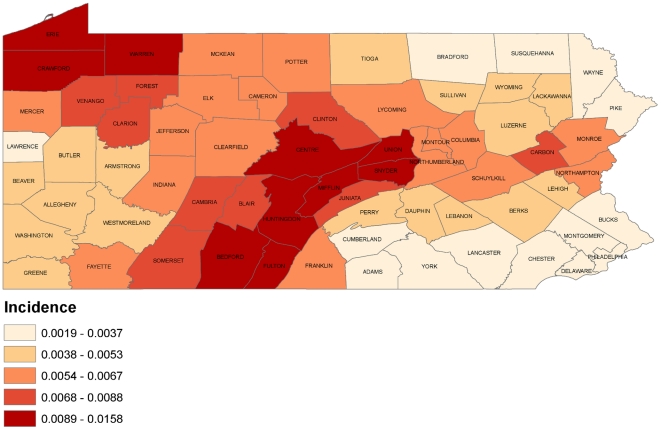
Cumulative incidence of six influenza seasons (2003–2009). Incidence presented using an Empirical Bayesian smoother to adjust for small populated counties.

**Figure 4:**
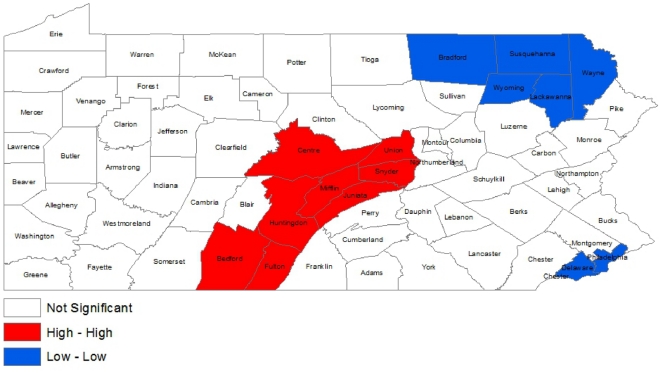
Spatial autocorrelation of 6-year cumulative incidence for 67 counties in Pennsylvania. Local spatial clusters were determined by the Local Indicator of Spatial Association (LISA) statistic. Regions designated as high-high (red) indicate clustering of similar values of higher incidence. Regions designated as low-low (blue) indicate clustering of similar values of lower incidence.

The Empirical Bayes smoothed cumulative incidence for the seasonal spatial distributions revealed clusters of elevated incidence in the Central and Northwestern portions of the state (Figure 3). The Southeastern and Northeastern regions of the state experienced consistently lower incidence for each season. The Moran's *I* statistic testing for global spatial autocorrelation of the cumulative incidence was 0.4959 (P = 0.07) indicating that neighboring counties have similar incidence, although not statistically significantly. In the local autocorrelation analysis, the central portion of the state was designated as “high-high” indicating clusters of similar elevated incidence (Figure 4). These counties included: Bedford, Centre, Fulton, Huntingdon, Juniata, Mifflin, Snyder, and Union. The areas of Philadelphia and Delaware counties and the Northeastern region were designated as “low-low” indicating these counties had local correlation of a lower incidence. Analysis of individual seasons demonstrated similar patterns as the cumulative six season cluster. Specifically, each individual seasonal cluster had a minimum of 3 counties similar to the six season cumulative cluster. Details can be found in Figure S2.

Descriptive statistics and results of the generalized linear model evaluating the relationship between membership in the elevated cluster and the predictor variables were presented in Table 3. The bivariate logistic regression found education>high school, age>64, total miles within the county, number of physicians, clinics, and hospitals, the rate of chronic lower respiratory disease, and precipitation associated with membership in the cluster (P<0.05). When including all predictors in a multivariable model, only mean monthly precipitation, age>64 and education>high school remained significant (P<0.05) (Table 4). For a one percent increase in the proportion of individuals aged over 64, the odds of membership in the cluster decreased adjusting for the other variables in the model (OR = 0.27, CI = 0.10, 0.73). Similarly the odds of membership in the cluster decreased for a percent increase in the proportion of individuals with more than a high school decgree (OR = 0.76, CI = 0.61, 0.95). An inch increase in monthly precipitation results in a 48% decrease in membership of the cluster (OR = 0.52, CI = 0.28, 0.94).

**Table 3:** Descriptive statistics and results of logistic regression model (Dependent variable are counties designated HIGH-HIGH in Moran's LISA cluster analysis, counties = 8).

Variable	Cluster = YesMean	Cluster = NoMean	Odds Ratio (OR)	P-value
**DEMOGRAPHICS** (N = 11)				
Household size	2.507	2.473	78.5707898	0.2940
Proportion of families w/1 child<18 years	0.4341	0.4394	0.00043812	0.5880
Proportion of families w/1 child<6 years	0.1745	0.1747	0.67139341	0.9850
Race (proportion white)	0.9647	0.9459	2.13165782	0.4890
Education>high school	0.3038	0.3778	2.6397E-08	**0.0319** *
Age>64	0.1448	0.1642	0.65856	**0.0308** *
Household income	35035	37467	0.99994100	0.3910
Population density per square mile	84.09	503.2	0.99133773	0.1010
Housing density per square mile	34.77	215	0.97170793	0.0795
Total road miles per area square miles	1.4892	3.1867	0.23015552	**0.0102** *
Highway miles per area square miles	0.08902	0.14375	0.00027550	0.1700
**HEALTH INDICATORS** (N = 8)				
Active physicians	71.75	619.7	0.9938191	**0.0144** *
Active physicians per 1000 persons	1.13	2.151	0.5231432	0.2010
Rural clinics and hospitals	1.688	4.766	0.56254	**0.0387** *
Rural clinics and hospitals per 1000 persons	0.03656	0.04483	0.01993	0.6730
ILI Sentinel Physicians	0.625	0.8305	0.8376960	0.6470
ILI Submissions	0.9023	0.6601	193.05980	0.1990
P&I mortality	2.40E-04	2.67E-04	0.967113	0.5200
Chronic lower respiratory disease	0.0004663	0.0005414	0.904023	**0.0423** *
**ENVIRONMENT** (N = 6)				
Elevation	1035.4	1227	0.999300	0.3370
Precipitation	3.174	3.501	0.591656	**0.0097** *
Minimum temperature	−1.711	−2.1358	1.209128	0.4438
Maximum temperature	8.963	8.502	1.252322	0.3945
Dew point	−2.172	−2.5419	1.488992	0.322
Absolute Humidity	868.9	848.5	1.00630	0.351

* Significance: P-value<0.05.

**Table 4:** Multivariable logistic regression model from bivariate results.

Variable	Odds Ratio (OR)	P-value
Age>64†	0.27	**0.0100** *
Education>high school†	0.76	**0.0148** *
Average precipitation (2003–2009)‡	0.52	**0.0319** *

* Significance: P-value<0.05.

† Interpreted as a 1% units.

‡ Interpreted as a 1 inch units.

A sensitivity analysis using the reduced data set, consisting of only cases with a collection date (N = 50421) was performed to assess whether the cases with missing dates displayed spatial and temporal biases. The sensitivity analyses reported limited differences in the spatial and temporal entities and did not influence membership in the cluster.

## Discussion

This was the first study to evaluate the spatial and temporal patterns of laboratory-confirmed influenza cases at the county level within a single state. There was evidence of spatial heterogeneity in the distribution of influenza in Pennsylvania. Using a test of spatial autocorrelation, local clusters of elevated incidence existed from Centre County in the central portion of the state extending to the Southern border counties of Fulton and Bedford. The extent of these elevated risks in this region persisted in each season. A combination of both demographic (age and education) and climatic variables (monthly precipitation) were significantly associated with membership in the elevated incidence cluster. Additionally, this study confirmed a previous finding that influenza B epidemics occur later in the season than influenza A [3], [8].

Time series analysis of weekly influenza surveillance identified by the World Health Organization and National Respiratory and Enteric Virus Surveillance System (WHO/NREVSS) collaborating laboratories for the entire United States and the Mid-Atlantic region (New York, New Jersey, Pennsylvania) showed similar timing of influenza A peaks compared to the PA-NEDSS data for most seasons under study [13]. Coinciding epidemic fade outs of influenza A and B were observed on a national level and within the Mid-Atlantic region from recent seasons: 2005/06 through 2008/09 (data not shown). Other regions of the country observed similar patterns of simultaneous declines. The concurrent weekly decline in reported cases for Pennsylvania may be the result of several factors including environmental drivers, host factors, diminished surveillance, and a small sample size. Changes in temperature and humidity as the winter shifts to spring may alter virus stability and influence patterns of crowding and host mixing leading to a simultaneous decline in incidence [6], [14]. Alternatively, diminished state surveillance as providers stop collecting and submitting specimens for influenza testing can lead to unreliable case estimates at the end of an epidemic producing an artifactual constraint on the epidemic time series. Seasonal time series encompassing longer surveillance periods are needed to control for the confounding effects of time in order to validate these findings.

This study is consistent with previous findings that the influenza B epidemic typically occurs later in the season than the influenza A epidemic. Finkelman et al. aggregated weekly incidence values over a nine year study period and demonstrated that influenza B temporally lags both the A/H3 and A/H1 subtypes in the Northern Hemisphere [8]. The degree of temporal similarity in peak epidemic timing of influenza A and B across the geographic scales (counties and continents) suggests that the factors driving the timing of the subtype epidemics could be similar within the Northern Hemisphere.

Comparing estimates of epidemic widths across seasons provides a measure of the speed and strength of the epidemic in the population. Estimates of the epidemic widths showed similarities to the peak durations observed among larger seasonal epidemics in Japan [11]. Differences in circulating influenza subtypes, particularly the introduction of new A/H3N2 antigenic variants in the Japan epidemics resulted in shorter peak activity periods [11]. This result was in contrast to seasons without new variants leading to epidemics that were smaller and displayed longer periods to attain peak activity. When comparing seasonal strain-specific information for the United States (not available for Pennsylvania), seasons dominated by the introduction of a new A/H3N2 virus (2004/05, and 2007/08) had shorter peak durations than 2005/06; an A/H3N2 season without a new antigenic variant season [15], [16]. In 2003/04 the A/Fujian/411/2002 A/H3N2 virus predominated and accounted for 88.8% of A/H3N2 isolates characterized which reported a less than optimal vaccine match [17]. In 2008/09, approximately 42% of all Pennsylvania cases were antigenically characterized by influenza B viruses. Nationally, influenza A cases were predominated by A/H1N1 (pre-novel H1N1) [13]. The 2008/09 season was not dominated by a new A/H3N2 variant, yet the epidemic length observed in this study from 2008/09 is not significantly different than the results from 2004/05 and 2007/08 when a new A/H3N2 antigenic variant appeared. In Pennsylvania, the first identified illness due to 2009 pandemic influenza A/H1N1 virus did not occur until the end of April and its appearance does not impact the data in this analysis. During the 2008/09 season the circulating A/H1N1 viruses were related to the vaccine component while less than 49% of the circulating influenza B viruses were related to the vaccine strain. Similar to the 2003/04 season, the vaccine mismatch among the influenza B virus may have contributed to the overall short epidemic duration. Nevertheless, the time period under study may not be representative of other influenza seasons and a longer time series is needed to confirm these results.

Discovery of the elevated incidence cluster in the central portion of the state warranted further investigation. The logistic model was designed to assess differences in characteristics for counties within and outside of the cluster with the specific intent of answering the question: what factors can explain the cluster of elevated incidence. Only age, education, and precipitation remained significant in the multivariable model.

The association of both age and education with membership in the cluster may be a reflection of differences in vaccination coverage between the counties. Poor vaccination coverage would create upward pressure on seasonal incidence rates and mortality [18], [19], [20]. Regional vaccination differences have been reported in urban and rural areas, age groups, and with increasing levels of education [21], [22]. According to the Behavioral Risk Factor Surveillance System, vaccination rates among the elderly (Age<65) in Pennsylvania only recently have approached the 70% Healthy People 2010 threshold [23]. The proportion of residents greater than 64 years and with more than a high school education was significantly lower among the counties in the cluster; which may suggest a lower vaccination rate in the cluster. Without available county-explicit data estimating seasonal influenza vaccination coverage, interpretation of the regional trends should proceed with caution.

Environmental factors including temperature and humidity have been long-associated as the driving force in the severity, spread and seasonality of influenza [6], [7], [24], [25], [26]. More recently, experimental and epidemiologic simulation studies have concluded that absolute humidity modulates influenza transmissibility leading to the observed seasonality in temperate climates [27], [28]. This report presented the results of multiple environmental factors including temperature, precipitation, dew point and absolute humidity. In this study we found a significant relationship with precipitation but not with absolute humidity, nor with any other environmental variables. The relationship of influenza incidence and precipitation has been inconsistent across studies as the associations tend to differ by country and influenza type [29], [30], [31], [32]. Associations of precipitation with the onset of influenza B have been observed, though these associations have not persisted with influenza A. For the climatic variables used in this analysis, the monthly results were averaged over the study period which is in contrast to previous studies that evaluated monthly differences in an effort to estimate the timing of influenza incidence or the onset of the influenza season which may have contributed to the contrasting results. There is no notable spatial correlation structure in the evaluation of influenza A and B in this dataset; thus, these comparisons cannot be made.

The passive surveillance system of PA-NEDSS creates reporting limitations. Even though Pennsylvania law mandates physicians, providers, hospitals, and laboratories to report specific disease data to PA-NEDSS, significant non-compliance has resulted in several types of ascertainment biases. First, the expected annual number of incident cases in the United States is estimated between 10%–20% which is substantially higher than the reported number of cases to PA-NEDSS [33], [34]. Many cases of influenza go undetected because the patient fails to seek treatment or is not tested for the disease. Spatial differences observed could also have been affected by testing practices of health care providers; those with access to free testing and a greater interest in influenza could result in a surge of testing. Inclusion of variables reflecting spatial location and submission history of influenza-like illness sentinel providers, who have access to free testing, was not associated with the cluster of elevated incidence; thus super testers are not likely to affect the spatial results observed.

The future of longitudinal data analysis within this data system is likely to be affected by the emergence of the H1N1 pandemic influenza subtype. Shifts in age distributions of pneumonia and influenza mortality have been noted in post-pandemic periods, which may have implications for the spatial distributions particularly in regions with younger populations [35]. Furthermore, there may be differences in the transmission parameters of the newly emerged influenza A subtype and the previous A/H1 subtypes in circulation resulting in further longitudinal distortions of the data. Despite these potential shortcomings, analysis of the transitional pandemic period remains an essential area for further exploration of these specific issues.

In conclusion, the epidemiology of influenza in Pennsylvania can be defined by a distinguishing spatial pattern. County level analysis revealed spatial patterns that would have been concealed by state-level analysis; a strength of this study. State and county public health officials should consider these findings in the utilization of human and economic public health resources to improve control strategies aimed at minimizing transmission through targeted vaccinations, directed hygienic advertisements, and informed surveillance. Additional research should focus on extending the analysis to the states of Maryland, Virginia, and West Virginia to determine if the spatial regime extends beyond the administrative borders.

## Methods

### Seasonal Cases

Laboratory-confirmed cases of influenza from 2003–2009 were obtained from Pennsylvania's National Electronic Disease Surveillance System (PA-NEDSS) managed by the Pennsylvania Department of Health [36]. The Pennsylvania National Electronic Disease Surveillance System is used to conduct surveillance of reportable diseases including influenza. The passive surveillance system began in 2003 and the system accepts PCR, culture and antigen tests from laboratories, hospitals, clinics, and individual providers in the form of online, electronic, paper or phone reports. Case reports are sent to NEDSS on average 5 days post-specimen collection date. The primary variables extracted from the database for this report included temporal attributes (sample specimen collection date, sample NEDSS report date), spatial attributes (subject home address latitude, and longitude, and zip code), influenza type, gender, reporting method, and date of birth.

For each season, the influenza season defined by the surveillance system ranged from October 1 through April 30 of the subsequent year. Cases were aggregated by week beginning with October 1 and each subsequent 7 days formed the next week. Specimen collection date was considered the date of diagnosis and used for all temporal and spatial analyses. If this date was not available (13% missing dates), a multiple imputation method used a Poisson regression to model difference between the specimen collection date and the NEDSS report data (100% complete data). Variables considered to be associated with incomplete reporting were included as covariates for the model (county, report method, season). To determine whether the cases with missing dates displayed spatial and temporal biases, a sensitivity analysis using a reduced data set of only cases with complete temporal properties was performed for all analyses.

### Statistical Analysis

The cumulative incidence for all six seasons was compared across counties. The total population of each county derived from annual population estimates of the US census served as the denominator [37]. For the presentation and spatial autocorrelation of the cumulative incidence by county, an Empirical Bayesian smoother was implemented to adjust for the inherent variance instability of the small incidence estimates given the small populations at risk [38], [39].

To assess differences in the duration of epidemics, a Gaussian distribution was fit to each epidemic using a non-linear least squares regression. Estimates of sigma (the width of the peak of the epidemic) for each epidemic were compared with 95% confidence intervals from each season.

Global spatial autocorrelation of the 6 year cumulative incidence was estimated by Moran's *I* statistic. This measure detects departures from spatial randomness; thus, a significant positive value would suggest that neighboring counties have statistically significantly more similar incidence than would be found among randomly selected pairs of counties. A significant negative statistic would indicate that neighboring counties have different incidence. Because the Moran's *I* statistic is a global test of spatial autocorrelation, the local indicator of spatial association (LISA) was used to detect local spatial clusters. Similar to the Moran's *I* statistic, the Local Moran statistic derives an estimate of significance based on a Monte Carlo permutation of the observations. The result is a thematic map which identifies the type of local clustering. Regions designated high-high or low-low indicated clustering of similar values; whereas, regions of high-low or low-high indicated a county was an outlier in the cumulative incidence relative to the neighboring counties [39], [40].

To identify predictors of an elevated incidence cluster from the LISA cluster analysis, a logistic regression modeled a binary outcome which was 1 if counties were in the high incidence cluster (N = 8) or 0 if not (N = 59). Each covariate was included separately in the model. A stepwise selection approach was used to identify significant predictors in the multivariable model Goodness of fit for the multivariable model was assessed using Akaike's Information Criteria. All p-values were two-sided based on a 95% significance level.

Covariates selected for the model reflected three broad categories: socio-demographics, health indicators, and the environment. Each variable has either previously displayed an association with influenza incidence and seasonality or could be a confounder in the relationship between spatial heterogeneity and the observed incidence [5], [6], [41], [42]. Social and demographic variable data obtained from the US Census included: age (proportion greater than 64), education (proportion greater than high school), race (proportion white), household income, population density (per square mile), and housing density (per square mile) [37]. Additional demographic variables summarizing the transportation networks in the region include highway miles (linear miles/total county area square miles), and total road miles (linear miles/total county area square miles) [43]. The health indicator variables obtained from the Area Resource File included county level data of active physicians (3 year mean 2005–2007/1000 persons), hospitals and rural health clinics (4 year mean 2003–2006 [Hospitals]+5 year mean 2003–2007 [Rural Health Clinics]/1000 persons), proportion pneumonia and influenza mortality (2003–2005 mean/population), and proportion chronic lower respiratory disease mortality (2003–2005 mean/population), also referred to as chronic obstructive pulmonary disease [44]. Distribution of influenza-like illness sentinel physicians (ILINet) and mean number of specimen submissions by provider were summarized for each county and included as a covariate. Climatic variables including precipitation (per 10 inches), temperature (in Celsius degrees) and dew point data were obtained for the study period (October–April) of each year and averaged over the time period [PRISM Climate Group, Oregon State University, http://www.prismclimate.org, created 4 Feb 2004]. Absolute humidity was calculated by converting the dew point temperature to vapor pressure and then divided by temperature multiplied by the gas constant for water vapor. Mean elevation (feet) was summarized for each county [45], [46], [47]. While human mobility between geographic regions has been shown to influence the spatiotemporal spread of influenza [5], this analysis was specifically concerned with risk factors for the elevated incidence cluster and not diffusion, thus this variable was not included in the model.

Statistical analyses were performed using the R statistical package (R Foundation for Statistical Computing, Vienna, Austria). Smoothing, and spatial autocorrelation were performed in STIS, (TerraSeer Inc., Crystal Lake, IL), and GeoDa (University of Illinois Urbana-Champaign, Urbana, IL). Institutional review board approval was obtained from the Pennsylvania Department of Health and the University of Pittsburgh.

## Supporting Information

Figure S1 **Individual Gaussian distribution results fitted to seasonal epidemics accompanied by the value of the standard error for the epidemic width.**
(TIFF)

Figure S2 **Local autocorrelation results specific for each influenza season (2003–2009).** Interpretation of the clusters are as follows: regions designated high-high (red) or low-low (blue) indicate clustering of similar values; whereas, regions of high-low (pink) or low-high (purple) indicate a county was an outlier in the cumulative incidence relative to the neighboring counties.
(TIF)
