# Supplementary figures and images for: Local Spatial and Temporal Processes of Influenza in Pennsylvania, USA: 2003–2009
Source: PLoS One. 2012 Mar 28;7(3):e34245. doi: 10.1371/journal.pone.0034245 (PMC3314628; doi:10.1371/journal.pone.0034245)

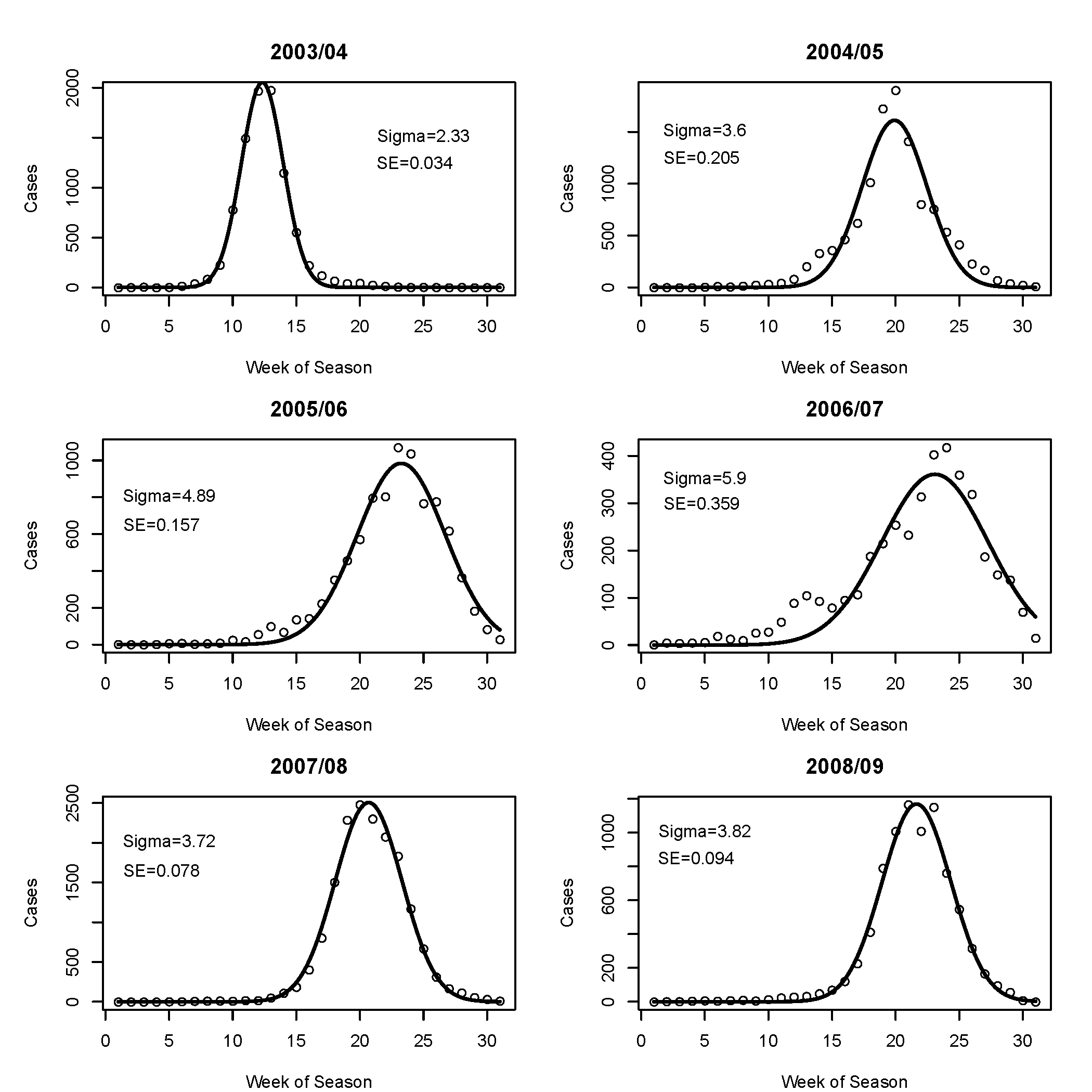

Supplement: Figure S1 — Individual Gaussian distribution results fitted to seasonal epidemics accompanied by the value of the standard error for the epidemic width. (TIFF) [file pone.0034245.s001.tiff]

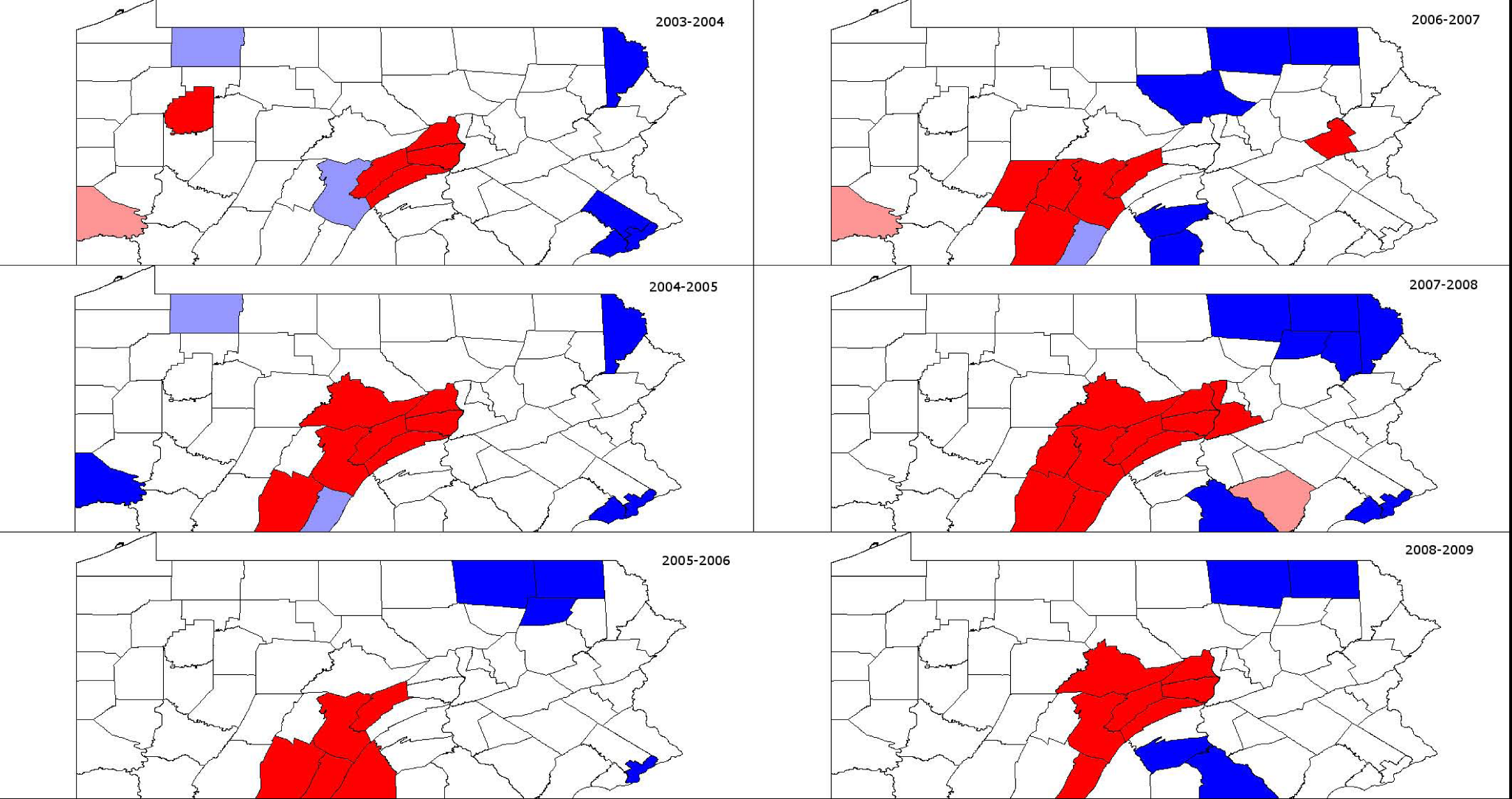

Supplement: Figure S2 — Local autocorrelation results specific for each influenza season (2003–2009). Interpretation of the clusters are as follows: regions designated high-high (red) or low-low (blue) indicate clustering of similar values; whereas, regions of high-low (pink) or low-high (purple) indicate a county was an outlier in the cumulative incidence relative to the neighboring counties. (TIF) [file pone.0034245.s002.tif]
